# Uptake of endocrine-disrupting chemicals by quagga mussels (*Dreissena bugensis*) in an urban-impacted aquatic ecosystem

**DOI:** 10.1007/s11356-018-3320-4

**Published:** 2018-11-03

**Authors:** Xuelian Bai, Kumud Acharya

**Affiliations:** Division of Hydrologic Sciences, Desert Research Institute, 755 E Flamingo Rd, Las Vegas, Nevada 89119 United States

**Keywords:** Endocrine-disrupting chemicals, Steroidal hormones, Pharmaceuticals, Invasive species, Bioaccumulation

## Abstract

Untreated organic contaminants in municipal wastewater, such as endocrine-disrupting chemicals (EDCs), have become a significant issue in aquatic ecosystems, particularly in freshwater bodies that receive wastewater discharge. This has raised concerns about the accumulation of EDCs in aquatic species via continuous exposure. This study evaluated the uptake of EDCs by quagga mussels (*Dreissena bugensis*), an invasive species in a water supply reservoir. The field sampling results showed that steroid hormones were not detected in the water samples, and only pharmaceuticals and personal care products were present (0.49 to 36 ng/L). Additionally, testosterone was the most abundant steroid in the mussel tissue (6.3 to 20 ng/g dry weight), and other synthetic chemicals (i.e., bisphenol A, triclosan, and salicylic acid) were also detected in the mussel tissue (24 to 47 ng/g dry weight). After being exposed to exogenous EDCs for 7, 21, and 42 days under controlled laboratory conditions, testosterone was not detected in the mussel anymore, but bisphenol A, triclosan, and salicylic acid were found at relatively high levels in the mussel tissue, although the concentrations did not increase over time. Overall, the study demonstrated the uptake of EDCs in quagga mussels, which suggests that this species can be used to reflect water quality deterioration in aquatic ecosystems.

## Introduction

Municipal wastewater treatment plants (WWTPs) often discharge organic contaminants at trace levels in the effluents, such as naturally occurring and synthetic hormones as well as pharmaceuticals and personal care products (PPCPs). These unregulated contaminants have become a significant concern in the environment because of their potential effects on the health of wildlife and humans at trace levels. These trace organic chemicals are frequently found at levels of parts per trillion to parts per billion in wastewater effluents (Jones et al. 2005; Lubliner et al. 2010; Miao et al. 2004; Miao et al. 2002; Soulet et al. 2002). Previous studies have also reported the presence of these contaminants at significant levels in surface waters worldwide (Ellis 2006; Kolpin et al. 2002; Lin and Reinhard 2005; Stan and Heberer 1997; Ternes et al. 2002). Therefore, the long-term ecological and human health effects, as well as the exposure routes, of untreated contaminants in aquatic ecosystems need to be evaluated.

Many emerging contaminants are documented to cause adverse health effects, such as endocrine disruption, in wildlife and humans (Brian et al. 2005; Brian et al. 2007). Fish and other organisms downstream from WWTPs are chronically exposed to endocrine-disrupting chemicals (EDCs) that may cause inappropriate sexual differentiation or development (Guillette et al. 1995; McLachlan 2001; Vajda et al. 2008). In addition to fish species, studies also reported the presence and uptake of EDCs in other aquatic species such as mollusks (Fernandes et al. 2011; Giusti and Joaquim-Justo 2013; Janer and Porte 2007; Scott 2012), mussels (Fernandes et al. 2010; Hallmann et al. 2016; Peck et al. 2007; Schwarz et al. 2017), algae (Bai and Acharya 2016; Bai and Acharya 2017; Maes et al. 2014; Zhang et al. 2014), and duckweed (Shi et al. 2010). Therefore, research activities attempt to identify the contaminants that can biomagnify in food chains and accumulate at harmful concentrations in higher trophic level organisms, including human beings. However, little information is available about the uptake of EDCs in bivalves, especially for synthetic chemicals that behave as xenoestrogens. The role that bivalves play in the accumulation and/or food web transfer of EDCs in aquatic ecosystems is still unclear.

Mussels have been used to indicate bioavailable concentrations of heavy metals and organic pollutants in aquatic environments. Mussels are efficient filter feeders that consume plankton and organic detritus, and they accumulate contaminants directly from the water column and from particulate matter (Richman and Somers 2005). The quagga mussel (*Dreissena bugensis*) is an invasive species that has spread through freshwaters across the United States, especially in the southwestern States. In southern Nevada, Lake Mead exhibits year-round warm temperatures, high calcium levels, and a lack of natural predators, all of which are strongly favorable conditions for the growth of quagga mussels. Additionally, Lake Mead represents a water supply reservoir that is affected by anthropogenic activities and used for drinking water, as well as recreation activities and a habitat for diverse wildlife species. Lake Mead is highly influenced by wastewater discharge from multiple WWTPs in the Las Vegas metropolitan area, where numerous untreated organic contaminants have been detected previously (Bai and Acharya 2017; Boyd and Furlong 2002; Rosen et al. 2010; Snyder and Benotti 2010). Studies conducted over the past two decades have shown continuous endocrine disruption in common carp (*Cyprinus carpio*) and largemouth bass (*Micropterus salmoides*) in Lake Mead (Bevans et al. 1996; Goodbred et al. 2015; Patino et al. 2003, 2015). Additionally, the Las Vegas Wash, constructed and naturally created wetlands that receive wastewater discharges and urban runoff, was found to have a significantly high estrogenicity response (Jones-Lepp et al. 2012). Therefore, quagga mussel may be exposed to EDCs in this aquatic ecosystem as well.

The goal of this study was to understand the uptake of EDCs by quagga mussels in an aquatic ecosystem using both field monitoring and laboratory experiments. The objectives of this research were to (1) measure ambient concentrations of a suite of EDCs in water samples and quagga mussels collected from different locations in Lake Mead, NV and (2) determine the bioaccumulation of EDCs by quagga mussels under controlled laboratory conditions via direct exposure to EDCs in the water or via food chain (i.e., algae feeding). This research provides much-needed insights into the occurrence, exposure routes, and environmental risks of EDC accumulation in invertebrates.

## Materials and methods

### Mussel and water sampling

**Fig. 1:**
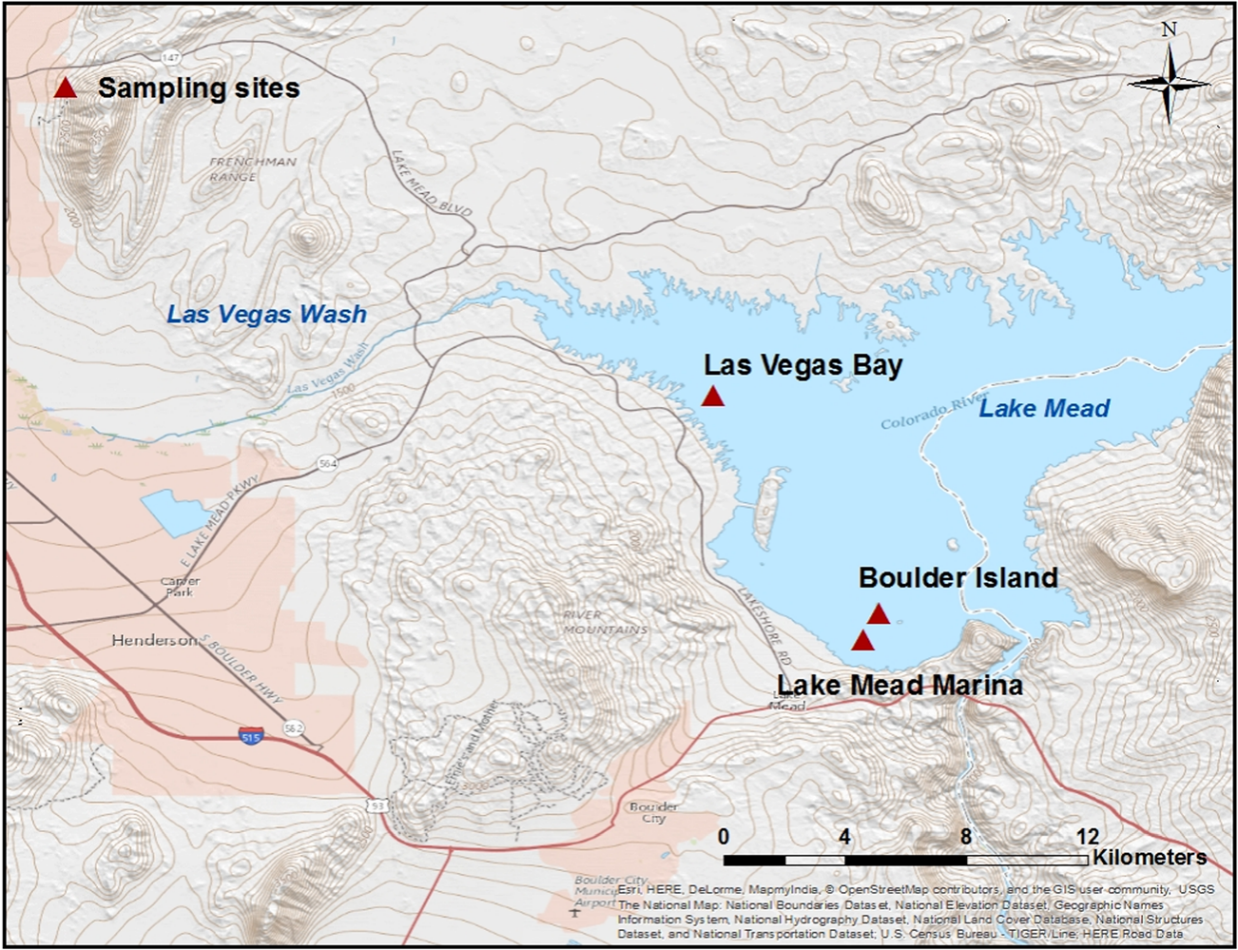
Map of sampling sites in Lake Mead, Nevada

Quagga mussels were collected from three locations in the Lake Mead area: Lake Mead Marina, Las Vegas Bay, and Boulder Island (Table 1 and Fig. 1). Mussels were collected at a 1-m depth from Lake Mead Marina and a 12-m depth from Las Vegas Bay and Boulder Island based on their availability at the different locations during the sampling season. The collected mussels were rinsed with lake water to remove debris, placed in ventilated containers filled with lake water, and then transported to the laboratory immediately. In the laboratory, adult mussels from each sampling site were rinsed with deionized (DI) water several times and deshelled immediately. Mussels larger than 12 mm were considered adults (Thaw 2013), and only adult mussels were selected for the following studies. Eventually, approximately 10 g (wet weight) of mussel tissue from each sampling site was obtained and kept frozen in a wide-mouth glass jar at − 20 °C. The mussel tissue was then shipped overnight on ice to the EPA certified Weck Laboratories Inc. (City of Industry, CA) for trace organic chemical analysis. An additional batch of mussels was collected from Lake Mead Marina only and kept in an aquarium tank filled with lake water to acclimate to the laboratory conditions at room temperature for a week. The aquarium was aerated and exposed to 12 h of light and 12 h of darkness to maintain normal algal growth. These mussels were used for the following bench-scale exposure experiments.

**Table 1:** Site description for water and quagga mussel sampling in Lake Mead

Site	Latitude (N)	Longitude (W)	Sample Date	Depth (m)
Lake Mead Marina	36.029306	− 114.772056	January 9, 2017	1
Boulder Island	36.037153	− 114.767529	February 14, 2017	12
Las Vegas Bay	36.101688	− 114.816596	February 14, 2017	12

Water was collected in duplicate simultaneously from the same sampling sites using 1-L amber glass bottles preserved with sodium azide (1 g/L) and ascorbic acid (50 mg/L). Water samples were kept on ice and transported to the laboratory immediately after collection. Water samples were kept at 4 °C in the laboratory until further chemical analysis performed. Additionally, bulk lake water was collected from Lake Mead Marina in 10 gal carboys and filtered using a 35-μm mesh filter to remove plankton, sediments, and large pieces of algae (Thaw 2013). The filtered water was stored in aerated buckets in the dark at room temperature for further experiments.

### Bench-scale exposure experiments

According to the field observations, the EDCs that were detected in the mussel tissue and all steroidal hormones were studied using a series of laboratory-based exposure experiments. Steroidal hormones are the most potent EDCs that accumulate in the mussel tissue and they can also cause adverse effects to other species within the food web. Although steroidal hormones were not frequently detected in the surface water or biomass, they are known as the most potent EDCs so far. For the exposure study, 30 adult mussels (size between 12 and 20 mm) were transferred from the aquarium tank to a 2-L beaker filled with the filtered, aerated lake water. Each beaker with 30 mussels was aerated and lightly covered with foil throughout the duration of the experiment (i.e., 7, 21, and 42 days) and kept at room temperature (~ 23 °C). During the experimental period, two studies were used to evaluate two potential exposure pathways: direct water exposure and food chain exposure (i.e., via algae feeding). For the direct water exposure treatment, the lake water was spiked with 50-μL EDC stock solution to reach the desired initial concentrations (Table 2) in the beginning of the experiment. To keep the water fresh and clean in each beaker, 1 L of water with mussel feces was disposed twice a week, and 1 L of fresh lake water spiked with the EDCs at the initial doses was added to refill the beaker. A freshwater green alga species, *Nannochloris sp.*, was used as a food source to keep the mussels alive and healthy throughout the experiments. Algal cultivation followed previous methods (Bai and Acharya 2016, 2017), and approximately 0.01 g (dry weight) of the alga was applied to feed the mussels on a daily basis.

**Table 2:**
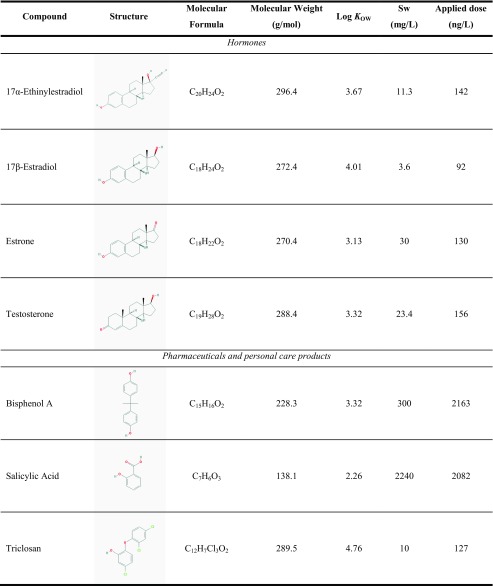
Properties of target EDCs used for laboratory-based experiments

For the food chain exposure treatment, every day before feeding, 5 μL of the stock EDC solution was added to the algae, which allowed binding of EDCs to the algal biomass, and the algae were then used to feed the mussels. Our previous findings showed that biosorption to algal cells was a major pathway for the uptake of hydrophobic compounds (Bai and Acharya 2016, 2017). The water changing process for this treatment followed the same process as the direct water exposure treatment, but no EDCs were spiked to the water to ensure that the EDCs applied were associated with the algal cells only. Five beakers were used for each treatment and for each experimental duration. For each treatment, at the end of the experiment (i.e., 7, 21, and 42 days), all 30 mussels from each beaker were collected and deshelled, and the tissue sampled from all five beakers was mixed together as one composite sample to obtain enough mass and to reduce the sample size. Each tissue sample (~ 10 g wet weight) was kept frozen at − 20 °C in a wide-mouth glass jar until further analysis could be conducted. A control using filtered lake water without EDC spiking was used to identify the effects of the laboratory conditions and the EDCs on the mortality of the mussels. The results showed no mortality throughout the study period even with EDC spiking.

### Chemical analysis

Standard chemicals of 17α-ethinylestradiol, 17β-estradiol, estrone, testosterone, bisphenol A, salicylic acid, and triclosan were purchased from Sigma-Aldrich (St. Louis, MO) with purity > 98%. The initial concentrations of the target EDCs were determined based on their reported levels in wastewater effluents and surface waters. The selected doses are approximately five times greater than the reported levels in wastewater effluents to ensure detection as well as avoid toxic effects on the mussels (Lubliner et al. 2010). The concentrations of the stock solution (in methanol) ranged from 3.7 to 86.5 mg/L. The physicochemical properties and initial doses of the compounds used in the study are summarized in Table 2. All analyses of the target compounds in the mussel and water samples were performed by the Weck Laboratories Inc. as described elsewhere (Bai and Acharya 2017). Briefly, the analytical method for water samples followed the EPA standard method for pharmaceuticals and personal care products (i.e., EPA 1694 M ESI-) and hormones (i.e., EPA 1694 M-APCI) using liquid chromatography tandem mass spectrometry (LC-MS/MS) (US EPA 2007), and an isotope spike was applied before sample pretreatment and extraction that was subject to the same analytical procedure. For the mussel tissue, the analytes were extracted using a quick, easy, cheap, effective, rugged, and safe (QuEChERS) method, which has been successfully used to determine pharmaceuticals in vegetables (Chuang et al. 2015) and fish (Lopes et al. 2012). Briefly, the mussel tissue was mixed with Na_2_EDTA solution and then added with acetonitrile and methanol. After adding anhydrous Na_2_SO_4_ and NaCl, the mixture was centrifuged at 2990 rpm for 10 min. The supernatant was collected, to which the d-solid-phase extraction sorbents (consisting of MgSO_4_, primary–secondary amine, C18, and graphitized carbon black) were added and centrifuged at 9240 rpm for 10 min. The mussel extracts were then analyzed for the target compounds using identical LC-MS/MS procedures to those used for the water samples.

## Results and discussion

### EDCs in water samples

The EDC concentrations in the water samples collected at the three study locations can be found in Table 3. Las Vegas Bay is the receiving waterway of municipal wastewater effluents. Boulder Island and Lake Mead Marina are further downstream of the Las Vegas Bay and expected to be less affected by wastewater discharge. However, these two locations are two of the most popular recreation sites at the lake, and recreational activities are considered the primary route for human exposure. Additionally, the nearby fisheries may also be influenced by these contaminants. Monitoring the contaminants using quagga mussels at these locations indicates the potential risks to other aquatic organisms, and even humans. The screening results showed that steroidal hormones were not found in any water samples. All of the PPCPs analyzed were detected in at least one of the sampling locations, except for iopromide. Bisphenol A, salicylic acid, and triclosan were found to be more abundant in the water samples compared with other analytes. The authors previously monitored over 200 wastewater organic contaminants in the Upper Colorado River watershed in Denver, Colorado, and reported that diclofenac, gemfibrozil, bisphenol A, and triclosan had median concentrations of 26.1, 32.3, 139, and 92.4 ng/L, respectively (Bai et al. 2018). The frequency of detection for diclofenac, gemfibrozil, bisphenol A, and triclosan was 40.1%, 42.5%, 50.9%, and 11.4%, respectively (Bai et al. 2018). Steroidal hormones were also found at low frequencies but relatively high concentrations at selected sampling sites in the Denver watershed: 17β-estradiol at 393 ng/L (11.4%), estrone at 112 ng/L (1.2%), and 17α-ethinylestradiol at 228 ng/L (9.6%). Additionally, triclosan has been detected at 2.6 and 8.0 ng/L in Lake Mead Marina and upstream of Las Vegas Bay, respectively (Bai and Acharya 2017). Similar monitoring studies have been done in Taihu Lake, China, where ibuprofen and diclofenac were detected at levels up to 65.3 ng/L (Xie et al. 2015).

**Table 3:** Concentrations of EDCs measured in water (ng/L) and mussel (ng/g dry weight) samples in Lake Mead

Analyte	Lake Mead Marina^a^	Boulder Island^b^	Las Vegas Bay^b^	MDL
Water (ng/L)				
Hormones				
17α-Ethinylestradiol	ND	ND	ND	0.56
17β-Estradiol	ND	ND	ND	0.31
Estrone	ND	ND	ND	0.20
Progesterone	ND	ND	ND	0.17
Testosterone	ND	ND	ND	0.14
Pharmaceuticals and personal care products				
Bisphenol A	1.2	30	22	0.27
Diclofenac	0.49	ND	1.5	0.26
Gemfibrozil	ND	ND	0.91	0.080
Ibuprofen	ND	0.55	1.5	0.39
Iopromide	ND	ND	ND	1.8
Naproxen	ND	ND	1.1	0.25
Salicylic Acid	17	28	36	0.86
Triclosan	ND	6.1	2.4	1.2
Mussel tissue (ng/g)				
Hormones				
17α-Ethinylestradiol	ND	ND	ND	5.0
17β-Estradiol	ND	ND	ND	5.0
Estrone	ND	ND	ND	5.0
Progesterone	ND	ND	ND	5.0
Testosterone	6.3	12	20	5.0
Pharmaceuticals and personal care products				
Bisphenol A	47	ND	ND	5.0
Diclofenac	ND	ND	ND	5.0
Gemfibrozil	ND	ND	ND	5.0
Ibuprofen	ND	ND	ND	5.0
Iopromide	ND	ND	ND	25
Naproxen	ND	ND	ND	5.0
Salicylic Acid	430	ND	ND	250
Triclosan	24	28	ND	10

^a^Sample collected at 1 m

^b^Sample collected at 12 m

*MDL*, method detection limit; *ND*, not detected

When comparing Lake Mead Marina and Boulder Island—which are the two adjacent sampling sites—the measured PPCPs appear to have higher concentrations in Boulder Island at 12-m depth (Table 3). This indicates that the contaminants are more persistent in the deeper water column, which may be because of low light exposure, low oxygen, and low microbial activities that inhibit the degradation of the contaminants. The persistence of EDCs in the deeper horizon may also cause a potential exposure risk for bottom-dwelling aquatic organisms. When comparing the Boulder Island and Las Vegas Bay sites sampled at the same depth (i.e., 12 m), more PPCPs were detected in the Las Vegas Bay water. Las Vegas Bay receives municipal wastewater effluent, surface runoff, and periodical flood water, all of which may be sources of the contaminants measured (Bai et al. 2018; Boyd and Furlong 2002; Rosen et al. 2010). Conversely, the contaminants can undergo natural attenuation and dilution as water flows into Lake Mead, resulting in lower detections (Bai and Acharya 2017). In a previous study performed at the same locations by the U.S. Geological Survey (Rosen et al. 2010), the highest concentrations of hydrophobic organic contaminants were found at a depth of 8 m in Las Vegas Bay. Rosen et al. (2010) also suggested that contaminants were generally confined to within 6 m of the lake bottom during the winter and spring, when Las Vegas Wash water sank to the bottom because of temperature and density contrasts between the Las Vegas Wash and Lake Mead water. The results of this study further demonstrate that many EDCs are present in the southern Nevada watershed. Therefore, the ecological impacts of these contaminants need to be fully evaluated despite the trace levels detected, and the vertical gradient of the contaminants needs to be monitored at different depths in the lake to assess the potential risks.

### Uptake of EDCs by quagga mussels

#### Steroids

For the quagga mussels collected from the field sites, testosterone was the only steroidal hormone that was detected (Table 3). Testosterone was not found in the water samples, but it was detected in the mussel tissue from all the three sampling sites at various levels listed in order from high to low: Las Vegas Bay > Boulder Island > Lake Mead Marina (Table 3). Many studies have reported the occurrence of steroidal hormones in mollusks—mainly testosterone and 17β-estradiol—but the origins are still unknown. Fernandes et al. (2010) found that the naturally occurring testosterone along the mussel reproductive cycle ranges from 0.1 to 1.4 ng/g, which is much lower than the concentrations measured in this study (i.e., 6.3 to 20 ng/g in Table 3). Scott (2012) reviewed most of the existing studies on vertebrate sex steroids found in mollusks so far, and he developed three hypotheses: (a) vertebrate steroids are found in mollusks because of the limitations of analytical procedures; (b) mollusks biosynthesize steroids themselves; and (c) mollusks accumulate steroids from the environment. Steroidal hormones in aquatic environments can be from wastewater effluents and animal feeding operations. Moreover, species such as fish are demonstrated to release steroids in urine and feces. Therefore, mussels are continuously exposed to exogenous steroids throughout their life cycle, and it is challenging to identify the origins of the steroids found in the mussel tissue. Furthermore, humans and animals usually release estrogens in biologically inactive conjugated forms (i.e., sulfate and glucuronide), which are also commonly found in aquatic environments (Bai et al. 2015; Bai et al. 2013). Interestingly, mollusks are known to be rich in the enzymes that hydrolyze the steroid conjugates, and therefore they are capable of taking up steroid conjugates from the environment and converting them to free steroids. All of these may be explanations for the occurrence of steroidal hormones in invertebrates. Although free steroids were not detected in the water samples in this study, it is speculated that free steroids and their conjugates might accumulate and transfer within the food web to cause adverse effects.

Testosterone was no longer detected in the mussels after they were exposed to EDCs under laboratory conditions for several weeks (Table 4). This is likely because the mussels may have released testosterone back to the water or esterified with fatty acids to stabilize the exogenous steroid. Similarly, Fernandes et al. (2010) stated that exposure to exogenous testosterone in the laboratory did not increase testosterone levels in the mussels and that the mussels likely excreted testosterone into the water. Mussels tend to esterify steroids with fatty acids and transform them into a more stable form that is resistant to metabolism (i.e., esterified steroids) (Scott 2012). Fatty acid esterification is a key mechanism that allows mussels to maintain their normal steroid levels following environmental exposure (Scott 2012). This pathway might have occurred in this study, but esterified steroids were not measured in the mussel tissue.

**Table 4:** Concentrations of selected EDCs measured in mussel tissue from laboratory-based exposure experiments (ng/g dry weight)

Analyte	7 days		21 days		42 days	
	Result	MDL	Result	MDL	Result	MDL
Direct water exposure						
Hormones						
17α-Ethinylestradiol	ND	0.67	ND	1.0	ND	0.55
17β-Estradiol	ND	0.67	ND	1.0	ND	0.55
Estrone	ND	0.67	ND	1.0	ND	0.55
Testosterone	ND	0.67	ND	1.0	ND	0.55
Pharmaceuticals and personal care products						
Bisphenol A	ND	0.56	ND	1.0	6.5	0.55
Salicylic Acid	ND	28	ND	51	ND	28
Triclosan	4.0	1.1	ND	2	ND	1.1
Food chain exposure						
Hormones						
17α-Ethinylestradiol	ND	0.75	ND	0.62	7.4	0.65
17β-Estradiol	ND	0.75	ND	0.62	ND	0.65
Estrone	ND	0.75	ND	0.62	ND	0.65
Testosterone	ND	0.75	ND	0.62	ND	0.65
Pharmaceuticals and personal care products						
Bisphenol A	26	0.89	ND	0.62	6.5	0.65
Salicylic Acid	830	44	ND	31	ND	33
Triclosan	ND	1.8	ND	1.2	ND	1.3

*MDL*, method detection limit; *ND*, not detected

Under laboratory conditions, the synthetic estrogen 17α-ethinylestradiol was detected at 7.4 ng/g after 42 days of exposure from the food chain (Table 4). 17α-Ethinylestradiol detection in the mussel tissue suggests that exogenous estrogens can be taken up by aquatic invertebrate species from the environment because 17α-ethinylestradiol is a synthetic estrogen that cannot form endogenously. 17α-Ethinylestradiol has been found in wild-caught mollusks at levels up to 80–130 ng/g of dry tissue weight (Liu et al. 2009; Lu et al. 2001). Naturally occurring estrogens were not detected in the mussel after 42 days of exposure, and therefore it appears that they either did not accumulate in the mussel or the estrogens were esterified and stabilized in the tissue. Of the naturally occurring estrogens, 17β-estradiol is the most frequently found in invertebrates, even though it was not detected in this study. Peck et al. (2007) reported that zebra mussels (*Dreissena polymorpha*)—which are freshwater bivalves—were susceptible to estrogen exposure, and when they were exposed to 5.5 ng/L of 17β-estradiol for 13 days, 17β-estradiol could accumulate in the mussel tissue at 840- and 580-fold in males and females, respectively. Additionally, testosterone and 17β-estradiol concentrations were found to increase dramatically over time in mollusks that were caged downstream of sewage treatment plants (Gust et al. 2010a; Gust et al. 2010b).

#### PPCPs

Bisphenol A, salicylic acid, and triclosan were found at relatively high levels in the mussel tissue based on the field observations (Table 3). Bisphenol A, salicylic acid, and triclosan can act as xenoestrogens because they have similar chemical structures to estrogens, and they may interfere with the endocrine systems of aquatic organisms. All of the contaminants detected are hydrophobic with log *K*_ow_ values ranging from 2.26 to 4.76 (Table 2), so they tend to be associated with the biomass in the aquatic ecosystem. According to the field monitoring study in Taihu Lake, China, roxithromycin, propranolol, diclofenac, and 17β-estradiol were found in mussels (*Anodonta*) with bioaccumulation factors of 406, 234, 70, and 59 L/Kg, respectively (Xie et al. 2015). All these findings demonstrated the bioaccumulation potential of selected wastewater contaminants in mussels in aquatic environments.

After being exposed to EDCs for 7, 21, and 42 days in the laboratory, the mussels were able to accumulate selected EDCs at different levels. For both the direct water exposure and food chain exposure studies, bisphenol A and triclosan were found in the mussel tissue at levels lower than the field observations, and salicylic acid from the food chain exposure study was measured at a higher concentration compared with the field observation (Tables 3 and 4). Moreover, bisphenol A and salicylic acid were measured at higher levels in the mussel tissue from the food chain exposure study compared with the direct water exposure study (Table 4), indicating that mussels may take up these compounds from the food source rather than directly from the water. Some mollusks are reported to be particularly susceptible to exposure to xenoestrogens such as bisphenol A, which can result in superfeminization of prosobranch snails (Jobling et al. 2003; Oehlmann et al. 2000). Triclosan was only detected after 7 days of exposure in the direct water exposure study (Table 4). This may be because triclosan is highly susceptible to light and it tends to photodegrade, and algae can increase its photodegradation rates (Bai and Acharya 2016, 2017). This results in the rapid dissipation of this compound in the aquatic environment and lower accumulation in the mussels. However, other studies have found triclosan in male common carp (*C. carpio*) from Las Vegas Bay to decrease sperm counts and induce vitellogenin (Jenkins et al. 2018; Leiker et al. 2009). Nonetheless, xenoestrogens may play a role in the reproductive physiology of aquatic organisms, but the adverse effects to aquatic invertebrates following exposure to xenoestrogens and exogenous steroids need to be further evaluated.

## Conclusions

This study evaluated the occurrence and uptake of a suite of steroidal hormones and other EDCs in quagga mussels, which are an invasive species found in the freshwater systems of the southwestern United States. The field sampling results showed that steroidal hormones were not detected in the Lake Mead water, and testosterone was found at relatively high levels in the mussel tissue from all of the sampling locations. Many other synthetic chemicals were detected in the lake water, but only triclosan, bisphenol A, and salicylic acid were found to be abundant in the mussels. Based on the laboratory exposure experiments, testosterone seemed to have the capability to be released back to water or esterified, so free testosterone was not found in the mussels after exposure to EDCs. Bisphenol A, salicylic acid, and triclosan were found to have accumulated at different levels in the tissue after exposure to EDCs. These results demonstrate that naturally occurring steroids and synthetic chemicals can accumulate in quagga mussel, and uptake via food chain is likely to be a primary pathway. This research provides useful information for understanding the potential of the widespread invasive species to accumulate wastewater organic contaminants, and has potential to be used as a biomonitor for endocrine disruption.
